# Usefulness of Preoperative Renal Scintigraphy for Treatment Planning before Endovascular Repair of an Abdominal Aortic Aneurysm with a Horseshoe Kidney: A Case Report

**DOI:** 10.70352/scrj.cr.26-0249

**Published:** 2026-09-05

**Authors:** Kota Kawada, Shinnosuke Okuma, Takeshi Kawamura, Takahide Yao, Tsubasa Yoshikawa, Erika Hanji, Toru Kameda, Takeshiro Fujii

**Affiliations:** 1 Division of Cardiovascular Surgery, Department of Surgery, Toho University Omori Medical Center, Tokyo, Japan; 2 Department of Nephrology, Faculty of Medicine, Toho University, Tokyo, Japan

**Keywords:** renal scintigraphy, horseshoe kidney, endovascular aortic repair

## Abstract

**INTRODUCTION:**

The optimal treatment strategy for abdominal aortic aneurysms associated with a horseshoe kidney remains controversial because of the complex renal vascular anatomy and the potential risk of renal function deterioration following accessory renal artery (ARA) occlusion. We report an exploratory case in which preoperative renal scintigraphy was used as an adjunctive functional assessment to assist treatment planning before endovascular aortic repair (EVAR).

**CASE PRESENTATION:**

A 47-year-old man was incidentally diagnosed with a 65-mm left common iliac artery aneurysm associated with a horseshoe kidney supplied by 8 ARAs. Preoperative dynamic renal scintigraphy demonstrated that the renal isthmus accounted for 24.5% of the total renal function. Because EVAR required the occlusion of 5 ARAs supplying the right kidney and renal isthmus, preoperative functional assessment suggested that sufficient renal function would likely be preserved despite partial ischemia of the isthmus, supporting the decision to perform EVAR. Postoperative renal scintigraphy demonstrated reduced perfusion of the isthmus but preserved overall renal function. Although the postoperative functional findings differed from the preoperative estimation, renal function remained clinically acceptable, and contrast-enhanced CT showed no evidence of an endoleak.

**CONCLUSIONS:**

This case suggests that preoperative renal scintigraphy may provide additional functional information beyond anatomical imaging and may assist treatment planning for selected patients with abdominal aortic aneurysms and horseshoe kidneys undergoing EVAR. However, the discrepancy between the preoperative functional estimation and the postoperative findings highlights the exploratory nature of this report, and further studies are required to clarify the clinical utility of this approach.

## INTRODUCTION

Open surgery for the treatment of an abdominal aortic aneurysm associated with a horseshoe kidney is technically challenging and carries a high risk of complications. Endovascular aortic repair (EVAR) has increasingly been adopted as an alternative treatment; however, the indications for EVAR remain controversial, owing to the risk of renal function deterioration and type II endoleaks attributed to the presence of accessory renal arteries (ARAs). Preoperative anatomical assessment using contrast-enhanced CT is essential for treatment planning, but it does not directly evaluate the functional contribution of individual renal segments supplied by ARAs. We report an exploratory case in which preoperative dynamic renal scintigraphy was used as an adjunctive functional assessment to support treatment planning before EVAR in a patient with an abdominal aortic aneurysm associated with a horseshoe kidney.

## CASE PRESENTATION

A 47-year-old man with hypertension underwent contrast-enhanced CT for evaluation of a pulmonary nodule, which incidentally revealed a 65-mm left common iliac artery aneurysm associated with a horseshoe kidney.

**Fig. 1:**
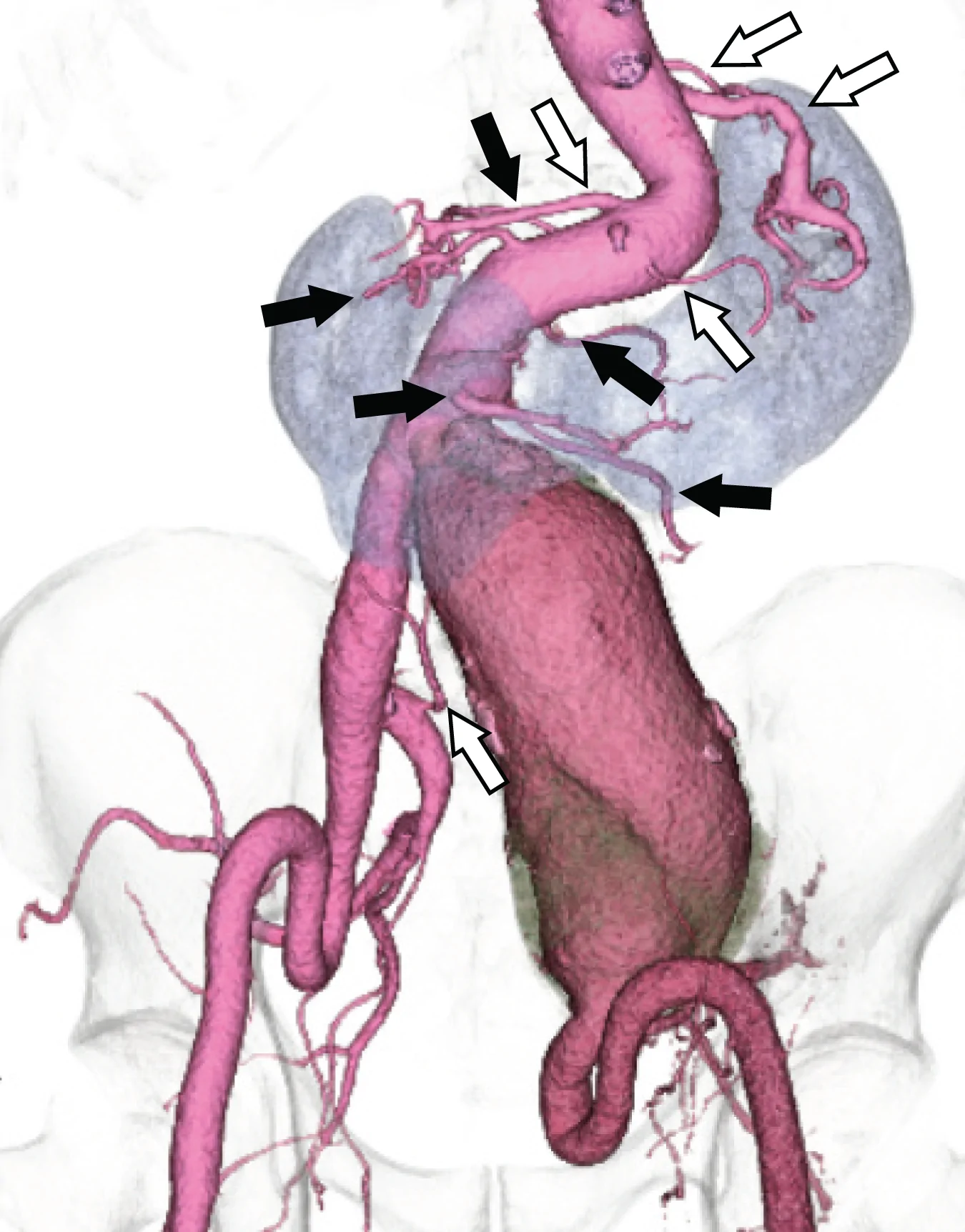
Contrast-enhanced CT revealed a left common iliac aneurysm with a maximum diameter of 65 mm, as well as numerous ARAs to be obstructed post-EVAR (EVAR; black arrows) and arteries that maintain blood flow post-EVAR (white arrows). ARAs, accessory renal arteries; EVAR, endovascular aortic repair

**Fig. 2:**
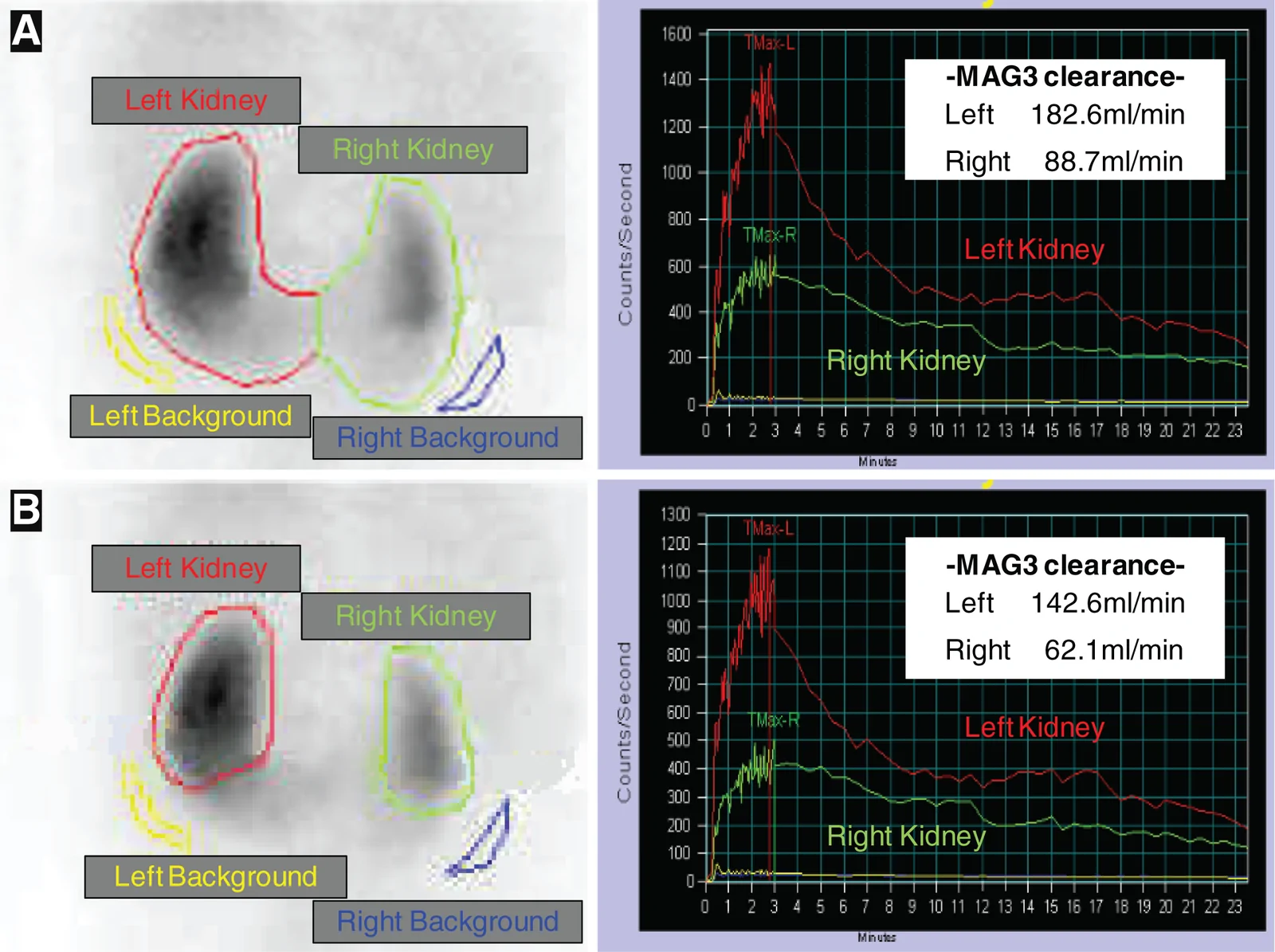
(**A**) Renal scintigraphy shows that bordering the isthmus of the horseshoe kidney, the MAG3 clearance of the left kidney was 182.6 mL/min, while that of the right kidney was 88.7 mL/min, indicating a left-sided predominance in renal function. (**B**) Excluding the isthmus of the horseshoe kidney, the MAG3 clearance was 142.6 mL/min for the left kidney and 62.1 mL/min for the right kidney. These results indicate that the MAG3 clearance of the isthmus was 66.6 mL/min. MAG3, mercaptoacetyltriglycine

The patient was 169 cm tall, weighed 70 kg, and had a painless pulsatile mass in the abdomen. Blood tests showed that creatinine was 0.91 mg/dL, and the estimated glomerular filtration rate (eGFR) was 71.2 mL/min. Contrast-enhanced CT revealed a left common iliac artery aneurysm with a maximum diameter of 65 mm and a horseshoe kidney supplied by 2 main renal arteries and 8 ARAs. Two ARAs supplied the left kidney, 1 supplied the right kidney, 1 supplied the isthmus from the right kidney, 3 originated directly from the abdominal aorta and supplied the isthmus, and 1 originated from the right internal iliac artery and supplied the isthmus (**Fig. 1**). Preoperative dynamic renal scintigraphy demonstrated a total mercaptoacetyltriglycine (MAG3) clearance of 271.3 mL/min, with left- and right-sided contributions of 67.3% and 32.7%, respectively, indicating left renal functional predominance (**Fig. 2A**). The renal isthmus accounted for 66.6 mL/min (24.5%) of the total MAG3 clearance, whereas the remaining renal parenchyma accounted for 204.7 mL/min (75.5%) (**Fig. 2B**). When performing artificial blood vessel replacement, the central anastomosis site is directly inferior to the renal isthmus of the horseshoe kidney; therefore, it is necessary to transect the same site. Considering the possibility of associated bleeding, urinary fistula, and ureteral damage, EVAR was considered the preferred treatment strategy. To ensure an adequate landing zone for the stent graft, occlusion of 5 ARAs was anticipated: 1 artery supplying the right kidney, 1 artery supplying the isthmus from the right kidney, and 3 ARAs arising directly from the abdominal aorta and supplying the isthmus (**Fig. 1**). Preoperative dynamic renal scintigraphy demonstrated that renal function was predominantly preserved outside the isthmus. These findings suggested that a substantial proportion of renal function would likely be maintained despite the anticipated loss of perfusion to the isthmus. Together with the anatomical findings on CT, the scintigraphic findings were incorporated into the preoperative treatment planning, and EVAR was selected.

**Fig. 3:**
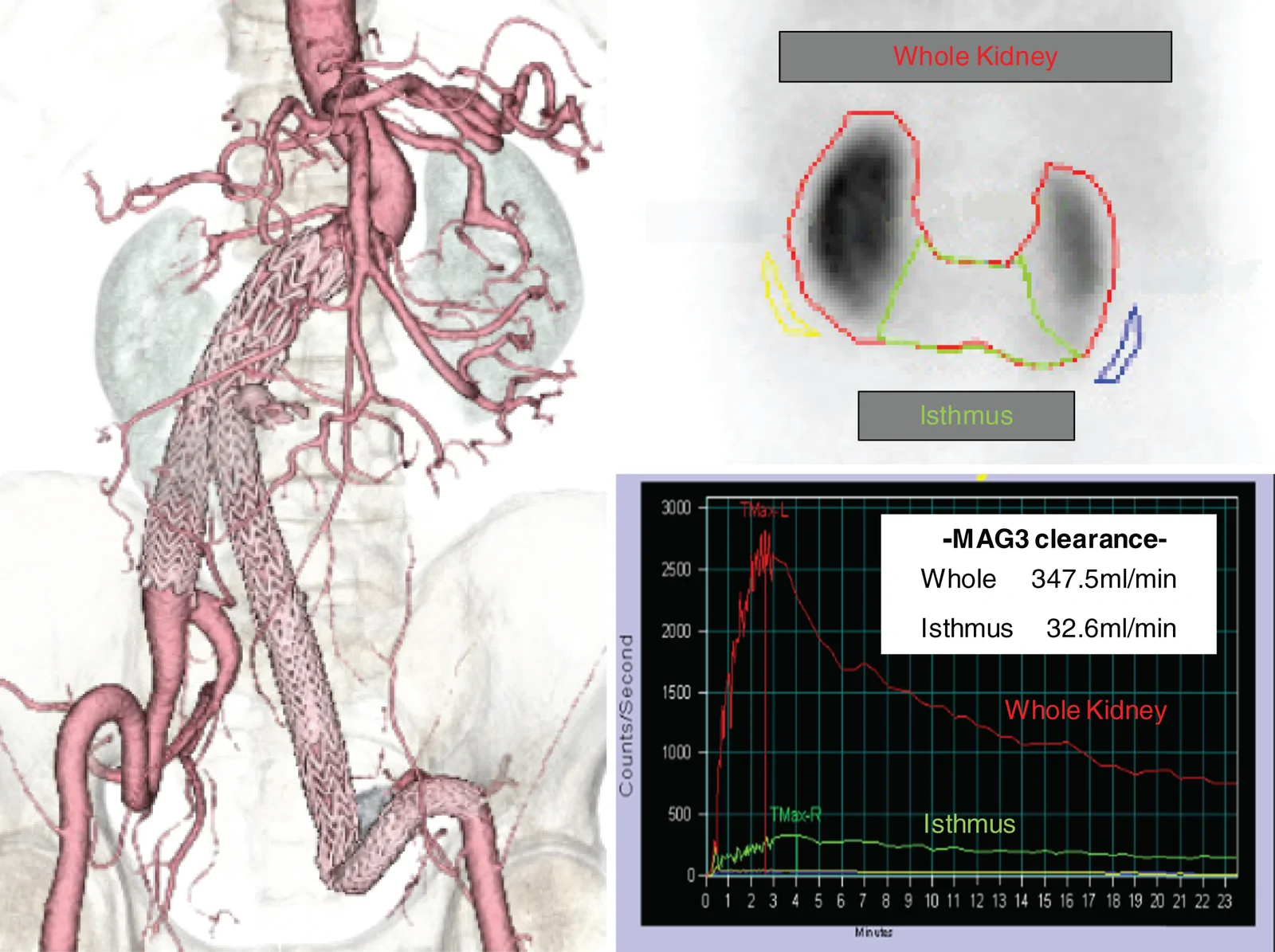
The stent graft was placed in the planned position, and no subsequent endoleak was observed. Renal scintigraphy showed that the post-EVAR MAG3 clearance of the isthmus was 32.6 mL/min, while the sum of the left and right kidneys excluding the isthmus was 314.9 mL/min. EVAR, endovascular aortic repair; MAG3, mercaptoacetyltriglycine

The EVAR was performed under general anesthesia, as follows: first, coil embolization was performed in the left internal iliac artery using one 10 mm × 40 cm, two 8 mm × 40 cm, and one 6 mm × 40 cm TargetXXL (Stryker, Kalamazoo, MI, USA), and one 5 mm × 15 cm TargetXL (Stryker, Kalamazoo) vascular coil; next, a GORE EXCLUDER main body, RLT281418J (W. L. Gore & Associates, Inc., Flagstaff, AZ, US), was inserted from the left common femoral artery just inferior to the right main renal artery, and because the contralateral leg required right external iliac landing, a PLC201000J was placed in the right external iliac artery; after ensuring overlap, a PLC141400 was extended proximally and deployed; next, for the ipsilateral leg, a PLC141400 was placed in the left common iliac artery; finally, intraoperative angiography was performed, which revealed good contrast in the kidneys with no endoleaks. The surgical time was 3 h 54 min. The postoperative course was uneventful. On the 4th day post-op, the serum creatinine level remained stable at 0.91 mg/dL, and the eGFR was 71.2 mL/min, with no evidence of postoperative renal dysfunction. Dynamic renal scintigraphy was performed on the 7th day post-op, and the MAG3 clearance of the renal isthmus had decreased to 32.6 mL/min (9.4%), whereas the remaining renal parenchyma accounted for 314.9 mL/min (90.6%) (**Fig. 3**). Although the postoperative functional distribution differed from the preoperative scintigraphic estimation, overall renal function was clinically preserved. A contrast-enhanced CT performed on the 8th day post-op demonstrated satisfactory stent graft positioning without evidence of an endoleak (**Fig. 3**). The patient was discharged from the hospital on the 9th day post-op.

## DISCUSSION

The management of abdominal aortic aneurysms associated with horseshoe kidneys remains challenging because of the highly variable renal vascular anatomy.^1)^ In particular, EVAR often requires intentional occlusion of ARAs to achieve an adequate proximal landing zone, raising concerns regarding postoperative renal function deterioration and the development of type II endoleaks.^2,3)^ Although anatomical classification systems and CT provide essential information regarding vascular anatomy, they do not directly assess the functional contribution of individual renal segments. This clinical challenge provided the rationale for incorporating preoperative renal scintigraphy into the treatment planning in the present case.

Previous reports have demonstrated the usefulness of preoperative anatomical assessment for estimating the functional impact of ARA occlusion. Dorffner et al. evaluated the perfusion territory of ARAs using selective CT angiography, whereas Handa et al. reported the utility of CT volumetry and the watershed sign for treatment planning. However, these approaches remain based primarily on anatomical imaging.^4,5)^ Renal scintigraphy provided functional information that could not be obtained from anatomical imaging alone. While contrast-enhanced CT accurately delineated the vascular anatomy and identified the ARAs that would be sacrificed during EVAR, it could not determine the functional contribution of the renal parenchyma supplied by each artery. In contrast, dynamic renal scintigraphy directly provides functional information regarding the contribution of individual renal segments, thereby complementing the anatomical assessment obtained from CT.

In the present case, dynamic MAG3 scintigraphy demonstrated that approximately 3-quarters of the total renal function originated from the renal parenchyma outside the isthmus, whereas the isthmus accounted for approximately 1-quarter of the total renal function. These functional findings complemented the anatomical information obtained from CT and contributed to the preoperative assessment of the potential impact of ARA occlusion. Although the postoperative functional distribution differed from the preoperative scintigraphic estimation, this discrepancy does not necessarily diminish the clinical value of renal scintigraphy in preoperative assessment. Rather than serving as a definitive predictive tool, renal scintigraphy provided additional functional information that was unavailable from anatomical imaging alone and contributed to treatment planning in this case.

Several factors may explain the discrepancy between the preoperative estimation and the postoperative findings. First, postoperative renal perfusion may have been preserved through collateral circulation that was not detectable on preoperative imaging. Second, functional adaptation of the remaining renal parenchyma after EVAR may have contributed to the greater-than-anticipated postoperative renal function. Finally, ROI-based functional analysis using renal scintigraphy is subject to methodological limitations, particularly in patients with horseshoe kidneys, because precise delineation of the renal isthmus is challenging and has not been standardized. Therefore, the functional estimation obtained in this case should be interpreted as an approximation rather than an absolute prediction.

The preservation of postoperative renal function despite the intentional occlusion of multiple ARAs raises the possibility that collateral circulation contributed to maintaining renal perfusion. Although no apparent collateral vessels arising from the lumbar arteries or other arteries were identified on postoperative CTA, microvascular collateral circulation beyond the spatial resolution of CT cannot be excluded. This mechanism may partially explain the discrepancy between the preoperative functional estimation and the postoperative findings.

Another concern associated with ARA occlusion is the potential development of type II endoleaks. In the present case, prophylactic embolization of the ARAs was not performed because the arteries originated from the normal aortic wall rather than from the aneurysm sac, and postoperative imaging demonstrated no evidence of an endoleak. Therefore, the necessity of prophylactic embolization should be determined on the basis of the individual vascular anatomy rather than routinely performed in all patients.^6–8)^

This report has several limitations. First, this is a single exploratory case, and the findings cannot be generalized to other patients with horseshoe kidneys. Second, the ROI-based analysis of the renal isthmus using dynamic renal scintigraphy has not been standardized, and the delineation of the isthmus is subject to operator-dependent variability. Therefore, the functional estimation presented in this report should be interpreted as an approximate assessment rather than a precise quantitative measurement.

Finally, treatment planning for patients with horseshoe kidneys should integrate anatomical findings, functional assessment, baseline renal function, and overall surgical feasibility, rather than relying on renal scintigraphy alone. Further studies involving larger numbers of patients are needed to validate the clinical utility of this approach.

## CONCLUSIONS

This case suggests that preoperative dynamic renal scintigraphy may provide complementary functional information beyond anatomical imaging and may support preoperative treatment decision-making in selected patients with an abdominal aortic aneurysm associated with a horseshoe kidney. However, renal scintigraphy should be considered an adjunct to anatomical assessment and overall clinical judgment, and its role in predicting postoperative renal function and determining treatment strategy requires further investigation in larger studies.

## DECLARATIONS

### Availability of data and materials

The datasets generated and/or analyzed during the current case report are not publicly available because they contain information that could compromise patient privacy. However, anonymized data are available from the corresponding author upon reasonable request.
